# Plasminogen Activator Inhibitor-1 Secretion by Autophagy Contributes to Melanoma Resistance to Chemotherapy through Tumor Microenvironment Modulation

**DOI:** 10.3390/cancers13061253

**Published:** 2021-03-12

**Authors:** Hong-Tai Tzeng, Jenq-Lin Yang, Yu-Ju Tseng, Chih-Hung Lee, Wei-Ju Chen, I-Tsu Chyuan

**Affiliations:** 1 Institute for Translational Research in Biomedicine, Kaohsiung Chang Gung Memorial Hospital, Kaohsiung 83301, Taiwan; jyang@adm.cgmh.org.tw (J.-L.Y.); wjc2020@cgmh.org.tw (W.-J.C.); 2 Department of Dermatology, Kaohsiung Chang Gung Memorial Hospital, Kaohsiung 83301, Taiwan; yuju311@cgmh.org.tw (Y.-J.T.); zieben@cgmh.org.tw (C.-H.L.); 3 Department of Internal Medicine, Cathay General Hospital, Taipei 10630, Taiwan; 4 Department of Medical Research, Cathay General Hospital, Taipei 10630, Taiwan; 5 School of Medicine, College of Medicine, Fu Jen Catholic University, New Taipei City 24205, Taiwan

**Keywords:** autophagy, plasminogen activator inhibitor-1, chemotherapy, melanoma, tumor microenvironment

## Abstract

**Simple Summary:**

Dysregulation of tumor autophagy is implicated in cancer progression and chemotherapeutic response. It is unclear how tumor autophagy modulates tumor microenvironment and thereby contributes to chemoresistance. In this study, we found that autophagy-dependent plasminogen activator inhibitor (PAI)-1 secretion contributed to melanoma resistance to mitoxantrone (MitoX), a chemotherapeutic agent clinically used for treating various types of cancers (but not melanoma), by shaping a pro-tumoral microenvironment. Disruption of autophagy activity or targeting PAI-1 pharmacologically reshaped a tumor-suppressive immune microenvironment and enhanced the susceptibility of melanoma to MitoX in vivo. Overall, the results show that targeting autophagy/PAI-1 axis can serve as a novel strategy to repurpose MitoX-based chemotherapy.

**Abstract:**

Autophagy plays a crucial role in maintenance of cellular homeostasis via intracellular signaling pathways, lysosomal degradation of selective cargo and mediating protein secretion. Dysregulation of autophagy has been implicated in tumorigenesis, tumor progression, and resistance to therapy. However, the mechanism of autophagy-dependent secretion involved in the responsiveness to chemotherapy is poorly understood. In this study, we showed that mitoxantrone (MitoX), a chemotherapeutic agent used for treating various cancers but not melanoma, induced autophagy in melanoma cells in vitro and in vivo. We also found that plasminogen activator inhibitor (PAI)-1 secretion by MitoX-induced autophagy modulated the pro-tumoral microenvironment. Attenuation of PAI-1 activity using a specific inhibitor, tiplaxtinin (TPX), or by targeting the autophagy gene, *Becn1*, induced efficient antitumor immunity, thereby overcoming the resistance to MitoX in vivo. Of note, the therapeutic efficacy of TPX was abolished in MitoX-treated *Becn1*-defective tumors. Collectively, our results demonstrate that tumor autophagy-dependent PAI-1 secretion impairs the therapeutic efficacy of MitoX and highlight targeting of tumor autophagy or its secretory cargo, PAI-1, as a novel strategy to repurpose MitoX-based chemotherapy for melanoma treatment.

## 1. Introduction

Melanoma is the most malignant skin cancer owing to its high metastatic potential. For advanced stage or unresectable melanoma, systemic therapies, including chemotherapy, immune checkpoint blockers, and target therapies with BRAF/MEK or KIT inhibitors, are necessary. With growing publications and trials, immune checkpoint blockers have become the mainstream therapy for metastatic melanoma. However, the safety and financial burden of immune checkpoint blocker therapy should be taken into consideration. Immune-related adverse events (irAEs) have been reported in various organs, including the skin, liver, gastrointestinal tract, and endocrine system (hypophysis, thyroid) [1], and found to occur in 16% of patients on nivolumab, 27% of patients on ipilimumab, and 55% of patients on combination therapy with both agents [2]. On the other hand, chemotherapy provides modest efficacy, but less adverse effects. Dacarbazine (5-[3,3-dimethyl-1-triazenyl]-imidazole-4-carboxamide [DTIC]) is the only FDA-approved chemotherapeutic agent for metastatic melanoma. However, DTIC monotherapy showed less than 20% objective response rate (ORR) in a combined analysis of randomized controlled trials [3]. Combination chemotherapy with DTIC and other chemotherapeutic agents, such as cisplatin, carboplatin, paclitaxel, BCNU, and tamoxifen, compared with monotherapy with DTIC, did not show significant survival benefit [4]. Therefore, DTIC acts as the standard treatment for metastatic melanoma despite its modest antitumor efficacy.

Autophagy plays various biological functions that mainly rely on targeting selected cargo for degradation via fusion with lysosomes [5]. Dysregulation of these functions is associated with cancer initiation and progression [6]. Recently, autophagic activity has also been shown to perturb the response to cancer treatment [7]. BRAF inhibitor-induced autophagy results in drug resistance and displays poor response to chemotherapeutic agents in melanoma [8]. Moreover, upregulated autophagy is associated with failure to response to chemotherapy in advanced melanoma patients [9]. Autophagy inhibition via the pharmaceutical inhibitor, hydroxychloroquine, or shRNA-mediated ATG5 gene silencing sensitizes melanoma cells to chemotherapy in a 3D culture system [9]. Collectively, these findings suggest that autophagy plays a cytoprotective role in melanoma cells against chemotherapy-induced cytotoxicity.

In addition to its well-characterized role in cellular waste degradation via fusion with lysosomes, autophagy has recently been implicated in extracellular release of soluble factors that modulate the tumor microenvironment. Beclin-1 targeting for inhibiting autophagy in melanoma cells has been shown to result in recruitment of natural killer (NK) cells in the tumor bed via C-C motif chemokine ligand 5 (CCL5) induction, thereby leading to tumor growth suppression [10,11,12]. It was also reported that autophagy-associated secretory profiling of melanoma cells and patient-derived serum samples showed interleukin (IL)-1β, chemokine C-X-C motif ligand 8, leukemia inhibitory factor, family with sequence similarity 3 member C, and dickkopf 3, which are involved in inflammation and tumorigenesis [13]. These results suggest that the autophagy-based secretory process plays a role in modulating the tumor microenvironment. However, how autophagy-mediated cargo secretion regulates the response to chemotherapy and modulates the tumor microenvironment remains poorly understood. Thus, in this study, we investigated the effects of autophagy inhibition on the response to chemotherapy (mitoxantrone [MitoX], an inhibitor of DNA Topoisomerase II and protein kinase C) and the underlying mechanisms in a melanoma mouse model. We identify that autophagy-dependent plasminogen activator inhibitor 1 (PAI-1) secretion is responsible for the chemoresistance of melanoma to MitoX. Blocking of PAI-1 bioactivity or targeting autophagy overcomes the resistance to MitoX and attenuates a pro-tumoral immune microenvironment.

## 2. Results

### 2.1. Autophagy Deficiency Induces a Tumor-Suppressive Microenvironment and Inhibits Tumor Growth In Vivo

To examine the effects of tumor autophagy on the response to chemotherapy, we first generated B16-F10 melanoma cells that stably expressed scramble (shC) and shRNA targeting Beclin1 (shBecn1#1 and shBecn1#2) by lentiviral transduction. Beclin1 downregulation in shBecn1#1 and shBecn1#2 cells was validated at the RNA and protein levels by quantitative reverse transcription-polymerase chain reaction (qRT-PCR) and immunoblotting, respectively (Supplementary Figure S1A). We further showed that SQSTM1 protein was accumulated and LC3 expression was reduced in Becn1-silenced cells, indicating a defect in the autophagic flux in Becn1-knockdown cells (Supplementary Figure S1B). Although similar in vitro proliferation rates were observed between the shC and shBecn1-silenced cells (Supplementary Figure S2A), Beclin1-defective B16-F10 cells (shBecn1), compared with shC cells, showed a significant reduction in tumor growth (*p* = 0.003, Supplementary Figure S2B), suggesting that autophagy in melanoma cells induced pro-tumorigenic activity in vivo. In fact, a significant decrease in tumor vasculature (CD31^+^, *p* < 0.001), cancer-associated fibroblasts (SMA^+^, *p* < 0.001), tumor-promoting M2 macrophages (Arg1^+^, *p* < 0.001), and infiltrating immunosuppressive regulatory T (Treg) cells (Foxp3^+^, *p* = 0.003) was observed in allografts derived from Beclin1-knockdown cells (shBecn1#1), as shown by immunohistochemistry (IHC) (Supplementary Figure S2C,D).

### 2.2. Plasminogen Activator Inhibitor (PAI)-1 Is Identified as a Potential Factor of Autophagy-Mediated Secretion

To identify the autophagy-associated soluble factor(s) that modulates the tumor microenvironment, the cytokine/chemokine profiles of conditioned media (CM) derived from shC and shBecn1#1 cells were analyzed using antibody array. Among the 111 different cytokine antibodies screened, PAI-1, a pro-tumorigenic factor found in several cancer types, including melanoma [14], was the predominant downregulated protein in Beclin1-deficient cells (Supplementary Figure S3A). The reduced PAI-1 level in the CM derived from Beclin1-knockdown cells was validated by enzyme-linked immunosorbent assay (ELISA) (Supplementary Figure S3B).

### 2.3. MitoX Treatment Induces Autophagy in Melanoma Cells

**Figure 1:**
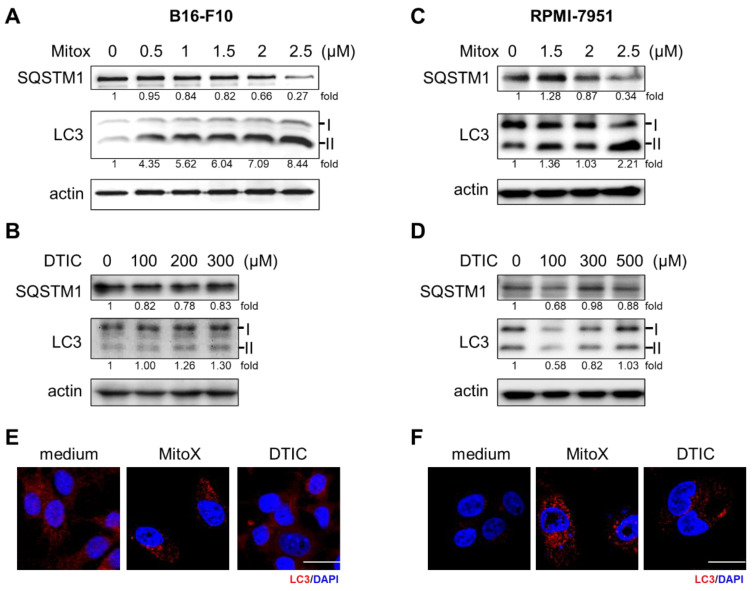
Mitoxantrone (MitoX) treatment induces autophagy in melanoma cells. (**A**,**C**) Murine B16-F10 and human RPMI-7951 cells were treated with MitoX at the indicated doses for 24 h. Cell lysates were harvested and subjected to immunoblotting using antibodies against SQSTM1 and light chain 3 (LC3). Actin was used as the loading control. (**B**,**D**) Immunoblotting analysis of SQSTM1 and LC3 in cells treated with dacarbazine (DTIC) for 24 h was performed. The numbers under the individual bands represented fold changes in LC3 and SQSTM1 expression on MitoX or DTIC treatment as compared to the control. (**E**,**F**) Confocal microscopy images of LC3 (red) and nucleus (blue) staining in cells subjected to chemotherapy were acquired. Scale bar, 20 μm. Whole western blots see Figure S8.

Since autophagy has been demonstrated to play a critical role in cancer progression and therapeutic response to chemotherapy [15], we examined whether MitoX, an anthracycline that is clinically used to treat several types of cancer, could increase the autophagic flux in melanoma cells. As shown in Figure 1, we observed gradual light chain 3 (LC3)-II accumulation and SQSTM1 degradation in B16-F10 cells 24 h after increasing the MitoX dose (Figure 1A). However, exposure to DTIC, the most common clinically used single chemotherapeutic agent for melanoma [16], did not interfere with autophagic activity (Figure 1B). We also observed similarly increased autophagic flux on MitoX treatment and no change in autophagic activity on DTIC treatment in RPMI-7951 human melanoma cells (Figure 1C,D). Accordingly, confocal microscopy analysis showed that MitoX treatment, but not DTIC stimulation, resulted in LC3 aggregation in both B16-F10 and RPMI-7951 cells (Figure 1E,F). These findings indicated an increase in autophagic influx in response to MitoX, but not DTIC, in vitro.

### 2.4. MitoX Promotes PAI-1 Secretion through Autophagy Induction

**Figure 2:**
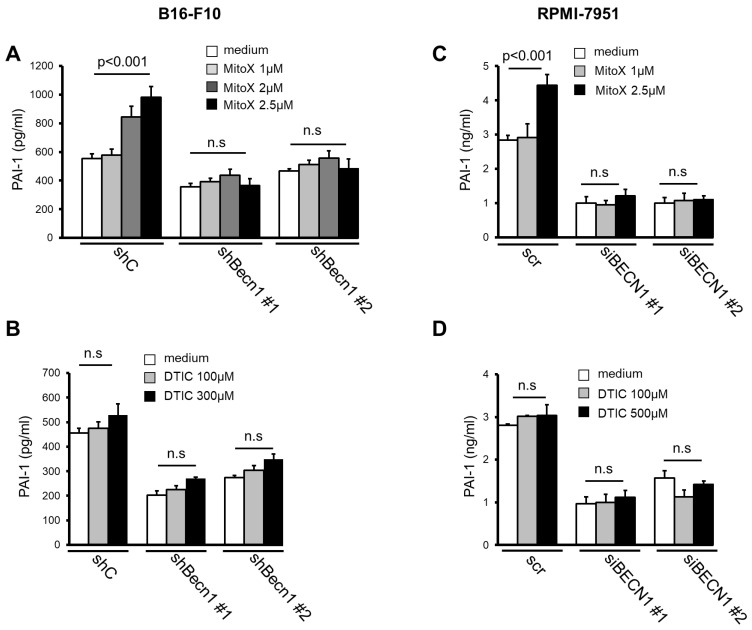
Mitoxantrone (MitoX) promotes plasminogen activator inhibitor (PAI)-1 secretion through autophagy. (**A**) Scramble (shC) or Becn1-silenced (shBecn1#1 and shBecn1#2) B16-F10 cells were treated with MitoX (1, 2, or 2.5 μM) for 24 h. Soluble PAI-1 levels in supernatants from the cells were determined by enzyme-linked immunosorbent assay (ELISA). (**B**) ELISA of PAI-1 concentration in CM from shC, shBecn1#1, and shBecn1#2 cells treated with dacarbazine (DTIC) was performed. (**C**) Scramble (scr)- or siBECN1-expressing (siBECN1#1 and siBECN1#2) RPMI-7951 cells were stimulated with MitoX (**C**) or DTIC (**D**) at the indicated doses for 24 h. Secreted PAI-1 amounts were analyzed by ELISA. *p* values were determined by two-tailed Student’s *t* test; n.s, not significant.

**Figure 3:**
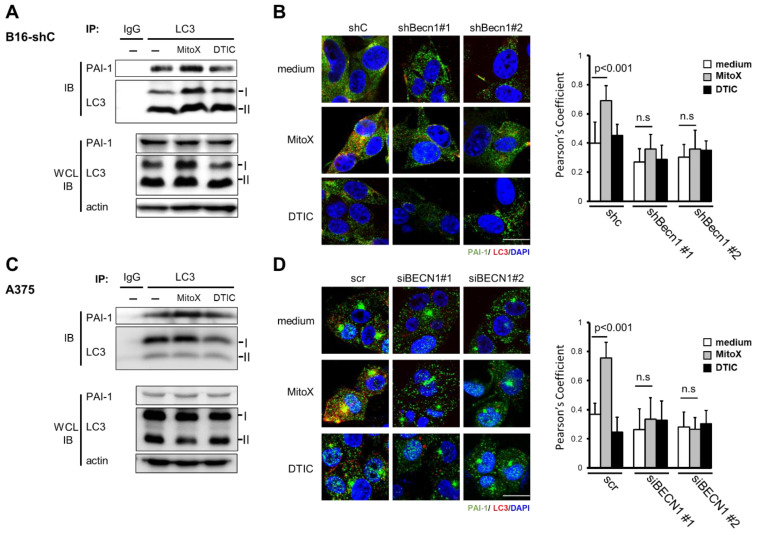
Plasminogen activator inhibitor (PAI)-1 interacts with light chain 3 (LC3) on mitoxantrone (MitoX) treatment. (**A**,**C**) Cells were treated with MitoX (2.5 µM for B16-F10-scramble (B16-shC); 0.5 µM for A375) or dacarbazine (DTIC, 300 µM for B16-shC; 500 µM for A375) for 24 h. Total protein lysates were immunoprecipitated using the anti-LC3 antibody. Immunoblotting was performed using anti-LC3 and anti-PAI-1 antibodies. WCL, whole cell lysates. (**B**,**D**) The representative confocal images of PAI-1 (green), LC3 (red), and nucleus (blue) staining in shC, Becn1-knockdown (shBecn1#1 and shBecn1#2) B16-F10 (**B**) and in scr, BECN1 silencing (siBECN1#1 and siBECN1#2) A375 cells (**D**) treated with MitoX or DTIC were acquired. Scale bar, 20 μm. Colocalization of PAI-1 and LC3 was quantified by counting 30 cells randomly. *p* values were determined by two-tailed Student’s *t* test; n.s, not significant. Whole western blots see Figure S9.

Next, we examined whether MitoX stimulation could induce PAI-1 secretion. The CM derived from MitoX-treated B16-F10 cells showed increased PAI-1 levels, as shown by ELISA (Figure 2A). Autophagy inhibition by Becn1 silencing abolished the increased soluble PAI-1 levels in MitoX-treated cells (Figure 2A), suggesting that MitoX promotes PAI-1 secretion through autophagy. In contrast, DTIC treatment showed no change in the secreted PAI-1 levels between the shC and Becn1-knockdown (shBecn1#1 and shBecn1#2) cells (Figure 2B). In addition, using RPMI-7951 cells and their BECN1-silenced derivatives (siBECN1#1 and siBECN1#2, Supplementary Figure S4), we observed similar effects of MitoX, but not DTIC, on the stimulation of PAI-1 release through autophagy as interruption of PAI-1 secretion was observe in BECN1-silenced cells (Figure 2C,D). Moreover, both B16-F10 and RPMI-7951 cells treated with chloroquine, a specific inhibitor of autophagy, impaired extracellular PAI-1 release (Supplementary Figure S5), supporting the notion that autophagy mediates PAI-1 secretion in melanoma cells. Additionally, PAI-1 localization in autophagosomes in response to MitoX stimulation was detected by the immunoprecipitation assay. Enhanced interaction between PAI-1 and LC3 was observed in MitoX-treated B16-F10-scramble (B16-shC) and A375 cells, whereas similar interaction between PAI-1 and LC3 was observed in DTIC-treated cells when compared with in control cells, (Figure 3A,C). Furthermore, confocal microscopic analysis showed a marked increase in the co-localization signals of PAI-1 and LC3 puncta in B16-F10 (shC) cells stimulated with MitoX, but not DTIC (Figure 3B). Similar results of PAI-1 and LC3 co-localization was observed in A375 human melanoma cells (Figure 3D). Of note, the MitoX-induced PAI-1 and LC3 co-localization signals were abolished in the autophagy-inhibited cells (B16-shBecn1#1 and shBecn1#2;Figure 3B; A375-siBECN1#1 and siBECN1#2, Figure 3D). Collectively, these results indicate predominant PAI-1 localization in autophagosomes in response to MitoX stimulation.

### 2.5. Autophagy Contrivutes to the Therapeutic Resistance of Tumor Cells to MitoX

**Figure 4:**
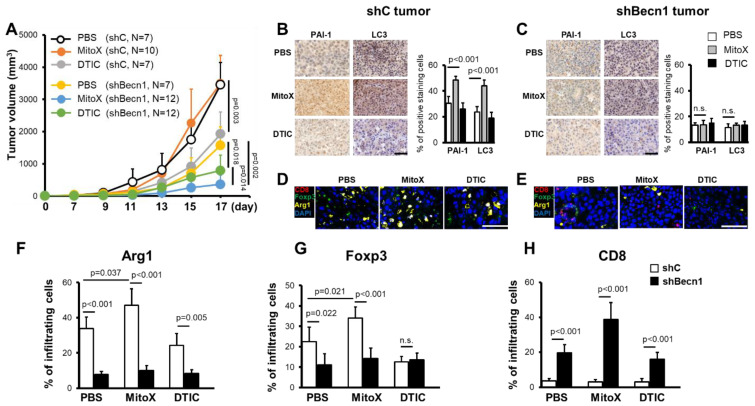
Chemoresistance of melanoma cells to mitoxantrone (MitoX) in vivo correlates with the pro-tumoral microenvironment. (**A**) C57BL/6 mice were subcutaneously inoculated with scramble-expressed (shC) or shBecn1#1 (shBecn1) B16-F10 cells. Phosphate-buffered saline (PBS), MitoX, or dacarbazine (DTIC) was intratumorally administered every day 7 days post-inoculation. Tumor volume was monitored at the indicated days. N denotes the number of mice per group. (**B**,**C**) Representative immunohistochemical images of plasminogen activator inhibitor-1 and light chain 3 staining in shC (**B**) or shBecn1 (**C**) tumor tissues, as described in (**A**), are shown. Positive-stained cells in shC and shBecn1 tumors were analyzed by defining regions followed by quantification using Phenochart and inForm^®^ software. Scale bar, 50 μm. (**D**,**E**) Multiplex immunofluorescent staining of shC (**D**) or shBecn1 (**E**) tumor sections was performed using anti-Arg1 (yellow), anti-Foxp3 (green), and anti-CD8α (red) antibodies, and counterstaining was performed using DAPI (blue). Scale bar, 50 μm. (**F**–**H**) Quantification of infiltrating Arg1^+^, Foxp3^+^, and CD8α^+^ cells in tumor regions was performed. Results were presented as the mean cell numbers of positive staining cells in five selected regions in each slide. *p* values were determined by two-tailed Student’s *t* test; n.s, not significant.

To elucidate whether autophagy-mediated PAI-1 secretion participates in the response to chemotherapy in vivo, we first subcutaneously injected C57BL/6 mice with shC cells and then treated them with MitoX or DTIC. In contrast to the cytotoxicity induced by MitoX and DTIC in cultured cells (Supplementary Figure S6), shC-tumor-bearing mice, compared with phosphate-buffered saline (PBS)-treated control mice, did not respond to MitoX insult, but DTIC treatment exerted a therapeutic effect (*p* = 0.003, Figure 4A). Interestingly, in contrast to Beclin1-proficient cells, which exhibited chemoresistance to MitoX, tumors derived from Beclin1-silenced cells showed sensitivity to MitoX when compared with those exposed to PBS (*p* = 0.002, Figure 4A). However, DTIC-based therapy still showed a therapeutic efficacy in shBecn1 tumors (*p* = 0.018; Figure 4A), which was comparable to that observed in scramble cell-derived tumors (45‒50% tumor suppression), suggesting that autophagy activity was independent of the efficacy of DTIC-based chemotherapy. Importantly, MitoX treatment displayed more effective tumor suppression in autophagy-deficient cells than did DTIC treatment (*p* = 0.014, Figure 4A). IHC images of shC-derived tumor sections showed enhanced PAI-1 and LC3 expression on MitoX treatment, supporting the in vitro findings that demonstrated autophagy and PAI-1 induction by MitoX, but not DTIC (Figure 4B). Accordingly, we observed low LC3 expression and reduced PAI-1 expression in response to MitoX treatment in Becn1-silenced tumor sections (Figure 4C). We further performed multiplex immunofluorescence to analyze the populations of the tumor immune microenvironment. Compared to the PBS-treated control tissues, MitoX-treated shC-tumor tissues showed increased levels of Arg1^+^ and Foxp3^+^ staining, which corresponded to pro-tumoral M2-subtype macrophages and Treg cells, respectively (*p* = 0.037 for Arg1^+^ cells, *p* = 0.021 for Foxp3^+^ staining; Figure 4F,G), whereas DTIC-treated tumor tissues showed modest reduction in the levels of these cells (Figure 4F,G). However, the frequency of tumor-killing CD8^+^ T cells was very low in all the examined tumor samples (Figure 4H). Consistent with the tumor suppression results, tumor-infiltrating M2 macrophages (Arg1^+^) were decreased in Beclin1-defective cell-derived tumors, compared with in scramble cell-derived tumors (Figure 4E,F). Additionally, reduction in Foxp3^+^ Treg levels was observed between the scramble and Beclin1-silenced tumors on PBS treatment, whereas no difference in Treg frequency was observed on DTIC treatment; however, Foxp3^+^ Treg infiltration was marked reduced in MitoX-treated autophagy-deficient tumors (Figure 4E,G). Of note, tumoricidal CD8^+^ T cell infiltration was enhanced in autophagy-deficient tumors, compared with in scramble-expressing tumors, on MitoX or DTIC therapy (Figure 4E,H). These results suggest that autophagy in melanoma cells contributes to chemoresistance to MitoX through induction of a pro-tumoral microenvironment.

### 2.6. Blocking of the Autophagy/PAI-1 Axis Overcomes Chemoresistance to MitoX

**Figure 5:**
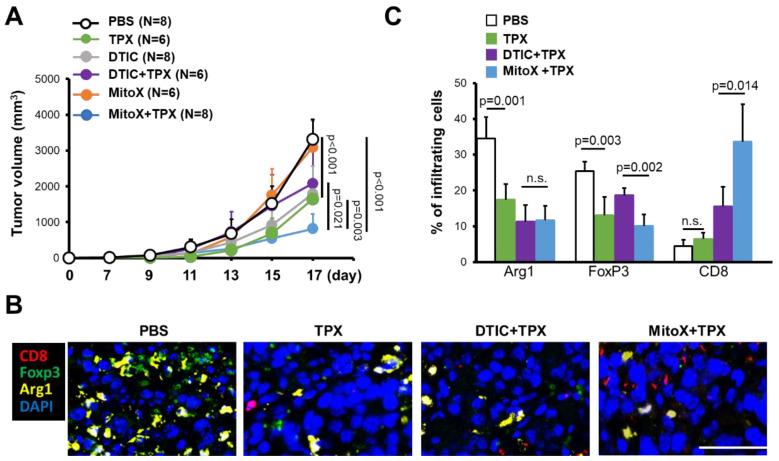
Pharmacological inhibition of plasminogen activator inhibitor (PAI)-1 overcomes chemoresistance to mitoxantrone (MitoX) and correlates with the anti-tumor microenvironment. (**A**) C57BL/6 mice were subcutaneously injected with scramble-expressed B16-F10 cells and then intratumorally administered with phosphate-buffered saline (PBS), MitoX, or dacarbazine (DTIC) daily or oral gavage administration of tiplaxtinin (TPX) every 2 days beginning at 7 days after inoculation. Tumor volume was measured at the indicated days. N denotes the number of mice per group. (**B**) Representative images of multiplex immunofluorescent staining in tumors subjected to PBS, TPX, DTIC and TPX or MitoX and TPX combination therapy were obtained. Arg1^+^ (yellow), Foxp3^+^ (green), and CD8α^+^ (red) cells were identified. Scale bar, 50 μm. (**C**) Quantification of staining positivity of cells in tumors, as described in (**B**), was shown. *p* values were determined by two-tailed Student’s *t* test. n.s, not significant.

**Figure 6:**
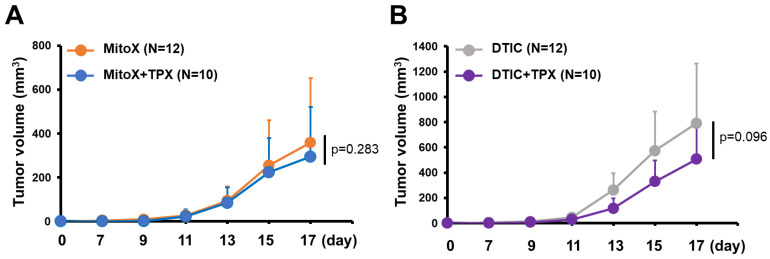
Autophagy/plasminogen activator inhibitor (PAI)-1 axis confers chemoresistance to mitoxantrone (MitoX). C57BL/6 mice bearing shBecn1 cell-derived tumor were treated with MitoX alone, MitoX and TPX (**A**), DTIC alone, or DTIC and TPX (**B**). Tumor volume was measured at the indicated days. N indicates the number of mice per group. *p* values were determined by two-tailed Student’s *t* test.

We then tested whether PAI-1 blocking in autophagy-proficient tumors could overcome chemoresistance to MitoX. Compared with that in the PBS-treated control group, therapeutic efficacy was observed in mice with shC-derived tumors treated with TPX, a specific inhibitor for blocking PAI-1 bioactivity [17] (*p* < 0.001, Figure 5A). Notably, shC tumor-bearing mice treated with MitoX in conjunction with TPX, compared with mice subjected to TPX or MitoX monotherapy, showed dramatic tumor growth inhibition (*p* = 0.003 for MitoX + TPX v.s. TPX; *p* < 0.001 for MitoX + TPX v.s. MitoX; Figure 5A). In contrast, DTIC and TPX combination therapy, compared with TPX or DTIC monotherapy, did not exert increased therapeutic benefits in tumor-bearing mice (*p* = 0.208 for DTIC + TPX v.s. TPX; *p* = 0.252 for DTIC + TPX v.s. DTIC, Figure 5A). These results suggest that PAI-1 plays a critical role in MitoX-elicited chemoresistance in melanoma. Strikingly, MitoX and TPX combination therapy exerted more effective tumor suppression in mice than DTIC and TPX combination therapy (*p* = 0.021, Figure 5A). Multiplex IHC images showed that mice receiving TPX therapy displayed reduced tumor-infiltrating Arg1^+^ M2 macrophages and Foxp3^+^ Treg cells, whereas the frequency of CD8^+^ cytotoxic T lymphocytes was not changed (Figure 5B,C). Additionally, pro-tumoral Treg cell infiltration was reduced, whereas CD8^+^ T cell infiltration was increased on MitoX and TPX combination treatment, compared with on DTIC and TPX combination treatment (Figure 5B,C). Of note, similar tumor growth inhibition was observed between shBecn1#1-derived tumor-bearing mice subjected to MitoX and TPX combination therapy and those subjected to MitoX monotherapy (*p* = 0.283, Figure 6A). However, shBecn1#1 tumor-bearing mice administered with DTIC and TPX, compared with those administered with only DTIC, displayed moderately reduced tumor growth, but this difference was not statistically significant (*p* = 0.096, Figure 6B). These results suggest that tumor autophagy-mediated PAI-1 secretion contributes to resistance to MitoX-based chemotherapy in melanoma.

## 3. Discussion

Accumulating studies have highlighted the critical role of tumor autophagy in mediating resistance to chemotherapy, but little is known about the role of autophagy-dependent secretion in the response to chemotherapy. Here, we demonstrated that MitoX-based chemotherapy, which has been widely used to treat various types of cancer (but not melanoma), induced autophagy-mediated PAI-1 secretion, thereby contributing to the resistance of melanoma to this chemotherapeutic agent. Extracellular PAI-1 release by MitoX-induced autophagy shaped the tumor microenvironment to support tumor cell survival. Targeting PAI-1 and/or autophagy markedly enhanced the susceptibility of melanoma to MitoX and induced anti-tumor immunity. Thus, we unravel a novel autophagy mechanism that is involved in chemoresistance.

Autophagy has been implicated in resistance to chemotherapy. Pharmorubicin-induced elevated autophagy and heme oxygenase-1 levels in triple-negative breast cancer cells mediate chemoresistance [18]. Pharmacological or genetic autophagy inhibition enhances breast cancer cell cytotoxicity in response to epirubicin [19]. Chemotherapy-induced cytotoxic aggregates promote autophagy-mediated clearance via the p62/SQSTM1-dependent mechanism to support tumor survival, thereby conferring chemoresistance [20]. Autophagic Forkhead box O3a (FOXO3a) degradation also contributes to tumor tolerance to chemotherapeutic stresses by suppressing FOXO3a-mediated pro-apoptotic signals [21,22]. Collectively, these findings suggest that tumor cells develop autophagy-dependent drug resistance via a cell-autonomous mechanism. However, our results showed that the resistance of melanoma to MitoX in vivo (but not in vitro, Figure 4 and Supplementary Figure S6) occurred due to tumor autophagic activity, since tumor autophagy inhibition by Becn1 silencing overcame chemoresistance to MitoX treatment and induced a tumor-suppressive immune microenvironment (Figure 4). Therefore, our findings indicate that tumor autophagy modulates the tumor microenvironment in response to chemotherapeutic stresses. In addition, it has been reported that PAI-1 acts as a positive regulator of autophagy [23,24], and reduced level of PAI-1 is associated with autophagy suppression [24]. In the current study, we showed that autophagy/PAI-1 axis contributes to chemoresistance. To clarify whether the increased sensitivity of the tumor to MitoX is due to the modulation of the tumor microenvironment, we treated shC and shBecn1-silenced cells (shBecn1#1 and #2) with MitoX and observed a similar cytotoxicity in these cells (Supplementary Figure S7A). Moreover, MitoX-induced cell death was blocked in both shC and shBecn1 cells with PAI-1 overexpression (Supplementary Figure S7B), suggesting a role for PAI-1 in chemoresistance.

The activity of tumor autophagy has been recently proposed to regulate an immune response in tumor-infiltrating immune cells. For example, Yamamoto et al. (2020) reported that autophagy routes surface major histocompatibility complex-I to lysosomes for degradation using the ubiquitin-binding receptor NBR1, thereby preventing T cell recognition of pancreatic cancer cells [25]. Autophagy also affects the therapeutic efficacy of immune checkpoint blockade by regulating programmed death-ligand 1 (PD-L1) levels. Targeting autophagy has been demonstrated to induce PD-L1 expression in p62/SQSTM1- and NF-κB-dependent manners [26]. Moreover, autophagy inhibition in melanoma cells has been demonstrated to enhance NK cell infiltration via CCL5 production through c-Jun activation [11]. These findings reveal that autophagy regulates tumor immune response via lysosomal degradation or intracellular signaling pathways. Recently, emerging evidence has shown that pro-inflammatory mediators, such as IL-1β, IL-18, and high mobility group box 1, are released via autophagy-dependent transport [27,28,29]. Of note, recent studies have focused on the effects of tumor-released autophagosomes on tumor immune suppression. Tumor-derived autophagosomes exert immunosuppressive activity by inducing PD-L1-expressing M2-like macrophages [30]. These extracellular autophagosomes also promote immunosuppression by modulating immunosuppressive functions of B cells and neutrophils [31,32]. In line with our observations, autophagy inhibition by Becn1 knockdown, which blocks autophagosome production, significantly induces a tumor-suppressive immune microenvironment. Our results showed that the increased Foxp3^+^ Treg cells, along with the scanty CD8^+^ T cells, were associated with MitoX-induced autophagy-dependent PAI-1 secretion (Figure 4, Figure 5 and Figure 6), suggesting that tumor autophagy modulates the tumor immune microenvironment in response to chemotherapy via the secretory pathway. Therefore, it may be interesting to determine whether PAI-1 serves as the cargo in tumor-released autophagosomes.

PAI-1 in the tumor microenvironment has been demonstrated to recruit monocytes and promote polarization of M2-type tumor-associated macrophages, thereby supporting tumor growth [33]. A previous study reported that cancer-associated fibroblasts stimulated with cisplatin secretes PAI-1 to promote esophageal squamous cell carcinoma progression and induce chemoresistance [34], indicating that the interplay between PAI-1 and the tumor microenvironment affects the chemosensitivity of the tumor cells. Consistently, our findings revealed that blocking of PAI-1 bioactivity using a small molecule inhibitor resulted in significant immunostimulation of the tumor microenvironment, thereby overcoming the resistance of the melanoma to MitoX insult (Figure 5). It has been demonstrated that PAI-1 plays a critical role in triggering macrophage migration through regulating the interaction between extracellular matrix and lipoprotein receptor-related protein (LRP) [35]. PAI-1 can also promote the recruitment and M2 polarization of macrophage to exert its pro-tumorigenic function by interacting with macrophage surface LRP1 and uPAR-uPA, respectively [33]. Therefore, it seems to be clear that PAI-1 exerts a direct effect on the regulation of macrophage motility and functionality. However, the role of PAI-1 in shaping T cell differentiation/function is still unclear. In the current study, we show that inhibition of PAI-1 in MitoX-treated tumors results in a decrease in M2 subtype macrophages and Tregs with a concomitant increase in CD8^+^T cells in tumor beds (Figure 5). Interestingly, secretion of IL-10 and TGF-β by tumor-infiltrating M2 macrophage can stimulate the immunosuppressive activity of Tregs [36]. Furthermore, the expression of enzymes, such as Arg1 and indoleamine 2, 3-dioxygenase from M2 macrophages also results in metabolic starvation of T cells, thereby enabling a strong immunosuppression [37]. Importantly, targeting PI3Kγ in myeloid cells by small molecule inhibitors shifts tumor-infiltrating macrophage toward the M1 subtype and enhances CD8^+^T cell population as well as the ratio of CD8+/Treg in a murine model of melanoma [38]. Thus, targeting tumor-infiltrating M2 macrophage may reshape the tumor immune microenvironment. Although the possibility of the direct effect of PAI-1 on T cell differentiation/function can-not be ruled out, we believe that the fluctuations in CD8^+^T and Treg populations in MitoX-treated tumor beds after PAI-1 inhibition at least in part results from phenotypic changes in infiltrating macrophages. We further demonstrated that tumor autophagy was responsible for PAI-1 secretion, highlighting another effect of autophagy regulation on chemotherapy efficacy.

## 4. Materials and Methods

### 4.1. Cell Culture

The murine melanoma cell line B16-F10 and the human melanoma cell lines A375 (ATCC^®^ CRL-1619™) and RPMI-7951 (ATCC^®^ HTB-66™) were purchased at the Bioresource Collection and Research Center (Hsinchu, Taiwan) and the American Type Culture Collection (Manassas, VA, USA), respectively. For generating stable Becn1-silenced cells, B16-F10 cells were transduced with a lentivirus that harbored shRNA targeting Becn1 (National RNAi core Facility, Institute of Molecular Biology, Academia Sinica, Taiwan). The cells were selected using puromycin (Sigma-Aldrich, St. Louis, MO, USA) for 2 weeks. For BECN1 silencing in RPMI-7951 cells, small interfering RNA (siRNA) for knockdown of human BECN1 (Ambion, Thermo Fisher Scientific, Waltham, MA, USA) were transfected into cells by using RNAiMAX regents (Thermo Fisher Scientific) for 48 h. All cells were maintained in Dulbecco’s Modified Eagle Medium (Gibco, Waltham, MA, USA), supplemented with 10% fetal bovine serum (Gibco) and 1% penicillin/streptomycin (Gibco), and cultured at 37 °C in 5% CO_2_.

### 4.2. Mouse Experiments

This animal study was reviewed and approved by the Institutional Animal Care and Use Committee at Kaohsiung Chang Gung Memorial Hospital (no. 2019091603), and performed in compliance with the relevant institutional guidelines and regulations. C57BL/6 mice were subcutaneously injected with B16-F10 melanoma cells (4 × 105 cells) that expressed scramble or shRNA targeting Becn1. Tumor volume was measured and calculated using the following equation: “V = (a × b^2^)/2”, where a and b are the tumor length and width, respectively. The mice were sacrificed 2-3 weeks after B16-F10 cell inoculation, and tumor tissues were resected, fixed, and prepared for IHC and multiplex immunofluorescent staining. For therapeutic treatment, DTIC (0.9 μg/dose, Sigma-Aldrich) or MitoX (1.5 μg/dose, Sigma-Aldrich) was administered daily via intratumoral injections beginning at 7 days post-inoculation during the entire experimental period. TPX (Axon MedChem, Groningen, The Netherlands) was administrated via oral gavage every 2 days. The mean tumor volume of subcutaneous implants in mice at day 17 was used for statistical comparison.

### 4.3. Immunofluorescence Assay

B16-F10, RPMI-77951 or A375 cells were cultured on coverslips and treated with MitoX or DTIC at the indicated doses for 24 h. The cells were fixed in 4% paraformaldehyde and then permeabilized with 0.5% triton X-100. After washing with PBS containing 0.05% Tween-20 (Sigma-Aldrich), the cells were incubated in blocking solution containing 5% normal goat serum in PBS and then incubated overnight with primary antibodies against PAI-1 (Novus Biologicals, Littleton, CO, USA) or LC3 (Abcam, Cambridge, UK) at 4 °C. The cells were then incubated with fluorescent dye-conjugated secondary antibodies at room temperature for 1 h in the dark. DAPI (Thermo Fisher Scientific) staining was performed to visualize the nucleus. The images were acquired and analyzed using FV10i confocal microscope and Fv31s-Sw software (Olympus, Tokyo, Japan), respectively.

### 4.4. IHC Staining

For IHC staining, tumor sections were dewaxed, rehydrated, and antigen-retrieved. After blocking, the slides were incubated overnight with primary antibodies against PAI-1, CD31 (Abcam), Arg1 (GeneTex, Irvine, CA, USA), α-SMA, (Abcam), or Foxp3 (eBioscience™, San Diego, CA, USA) at 4 °C. Peroxidase activity was visualized using the diaminobenzidine tetrahydroxychloride solution. The sections were counterstained with hematoxylin. For multiplex immunofluorescent staining, tumor-bearing mice subjected to chemotherapy were sacrificed to collect tumor samples. The de-paraffinized slides were fixed and antigen-retrieved. Following incubation with the blocking buffer, primary antibodies, including anti-Arg1 (1:10,000 dilution, GeneTex), anti-Foxp3 (1:8000 dilution, BioLegend, San Diego, CA, USA), and anti-CD8α (1:5000 dilution, Cell Signaling Technology, Danvers, MA, USA), were sequentially applied at 25 °C for 1 h. The slides were then incubated with horseradish peroxidase-conjugated secondary antibody (Akoya Biosciences, Marlborough, MA, USA). Tyramide-signal amplification was performed for the detection of antigen-expressing cells using Opal fluorophores (Arg1 Opal 570, FOXP3 Opal 520, CD8 Opal 690), according to the manufacturer’s instructions. Images were acquired using a Vectra Polaris instrument and analyzed by using the Phenochart and inForm^®^ software (Perkin Elmer, Waltham, MA, USA). To define the staining positivity, the mean intensity of six selected gates in an individual tissue slide stained with nonreactive isotype control antibodies was calculated. Quantification of positive-staining cells was carried out by counting positive-staining cells in five selected regions in each individual tissue slide and was normalized to that of control cells.

### 4.5. Immunoprecipitation

MitoX- or DTIC-treated cells, along with control cells, were harvested for 24 h. After washing with PBS, cell proteins were extracted by incubation with the lysis buffer. Immunoprecipitation assays were performed by adding protein A agarose bead (Merck Millipore, Burlington, MA, USA) to the cell lysates and incubating them overnight with specific antibody against LC3 (MBL) at 4 °C. The immune-complexes were separated by SDS-PAGE and detected by immunoblotting using anti-LC3 (Abcam) and anti-PAI-1 (Novus Biologicals, Littleton, CO, USA) antibodies.

## 5. Conclusions

In conclusion, our results demonstrate that autophagy in melanoma cells mediates PAI-1 secretion to induce immunosuppression of the tumor microenvironment, leading to chemoresistance. Targeting the autophagy/PAI-1 axis promotes the switch from immunosuppressive profiles to antitumor phenotypes of tumor-infiltrating immune cells and restores sensitivity to chemotherapy. These findings provide a rationale for the employment of inhibition of the autophagy-dependent secretion pathway as a therapeutic strategy to repurpose the clinically used chemotherapeutic agents for treatment of melanoma.

## Data Availability

The datasets used and/or analyzed during the current study are available from the corresponding author on reasonable request.

## Supplementary Materials

The following are available online at https://www.mdpi.com/2072-6694/13/6/1253/s1, Figure S1: Autophagy-deficient melanoma cells are established, Figure S2: Autophagy deficiency suppresses tumor progression, Figure S3: Plasminogen activator inhibitor (PAI)-1 is identified as a cargo candidate for autophagy-mediated secretion, Figure S4: Validation of Beclin 1 expression in RPMI-7951 cells, Figure S5: Inhibition of autophagy by chloroquine (CQ) blocks PAI-1 secretion, Figure S6: Cell viability in response to chemotherapy, Figure S7: Cytotoxicity of mitoxantrone (MitoX)-treated Beclin1 knockdown and PAI-1 expressing cells, Figure S8: whole western blots of Figure 1, Figure S9: whole western blots of Figure 3. Figure S10: whole western blots of Supplementary Figures.
