# Supplementary material for: Plasminogen Activator Inhibitor-1 Secretion by Autophagy Contributes to Melanoma Resistance to Chemotherapy through Tumor Microenvironment Modulation
Source: Cancers (Basel). 2021 Mar 12;13(6):1253. doi: 10.3390/cancers13061253 (PMC7999393; doi:10.3390/cancers13061253)
Supplement: Supplementary file 1 [file cancers-13-01253-s001.pdf]

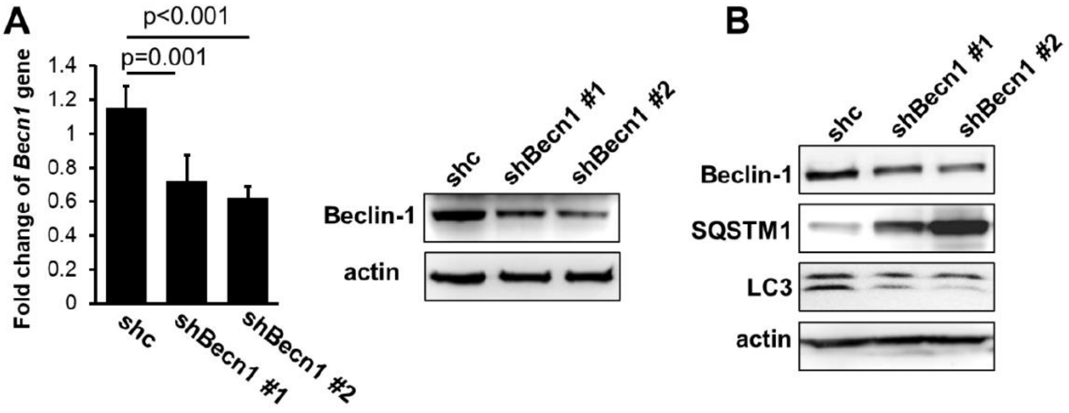

Figure S1. Autophagy-deficient melanoma cells are established. (**A**) B16-F10 cells were transduced with lentiviral-based shRNA targeting scramble (shC) or Becn1 gene (shBecn1#1 and shBecn1#2), and puromycin-resistant clones were selected. Becn1 RNA and protein expression levels in two clones were analyzed by quantitative reverse transcription-polymerase chain reaction (left panel) and immunoblotting (right panel), respectively. (**B**) SQSTM1, Beclin1 and LC3 levels in stable cells were determined by immunoblotting.

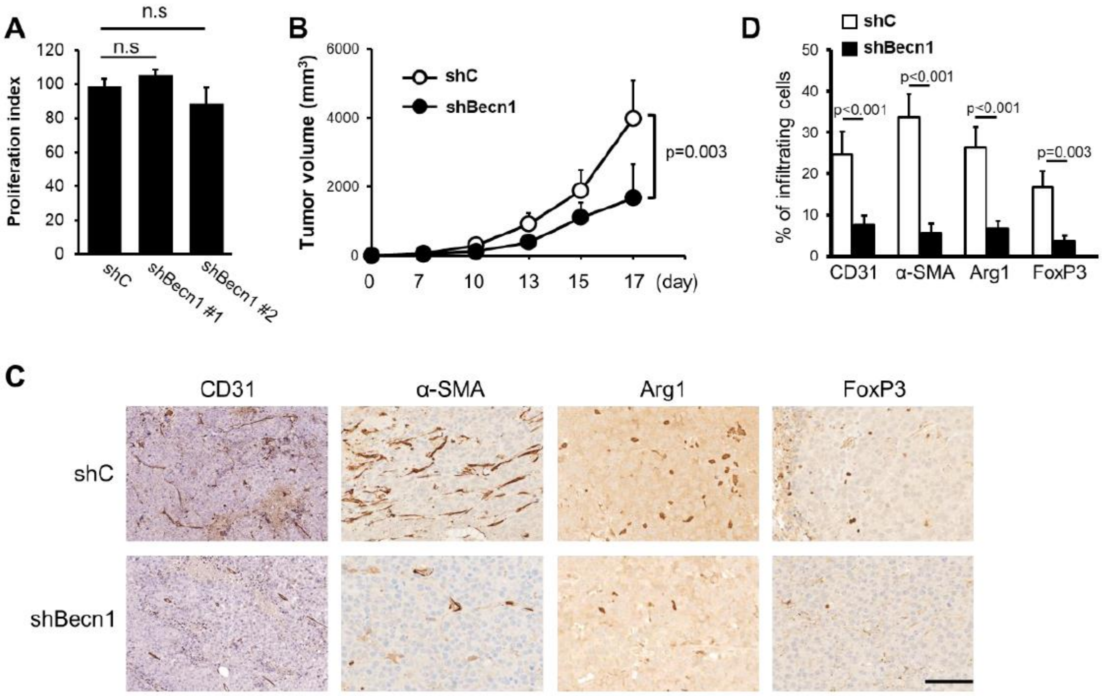

Figure 2. Autophagy deficiency suppresses tumor progression. (**A**) Proliferation of cultured B16-F10 cells (scramble [shC], shBecn1#1, and shBecn1#2) was measured by CCK-8 assay. (**B**) shC and shBecn1#1 B16-F10 cells were subcutaneously injected into C57BL/6 mice, and tumor volume was monitored at the indicated time points. (**C**) Immunohistochemistry of CD31, α-SMA, Arg1, and Foxp3 was performed for tumor sections, as described in (**B**). Scale bar, 50 μm. (**D**) Positive-stained cells in shC and shBecn1 tumors were calculated by defining regions and analyzed by using Phenochart and inForm® software. *p* values were determined by two-tailed Student's *t* test.

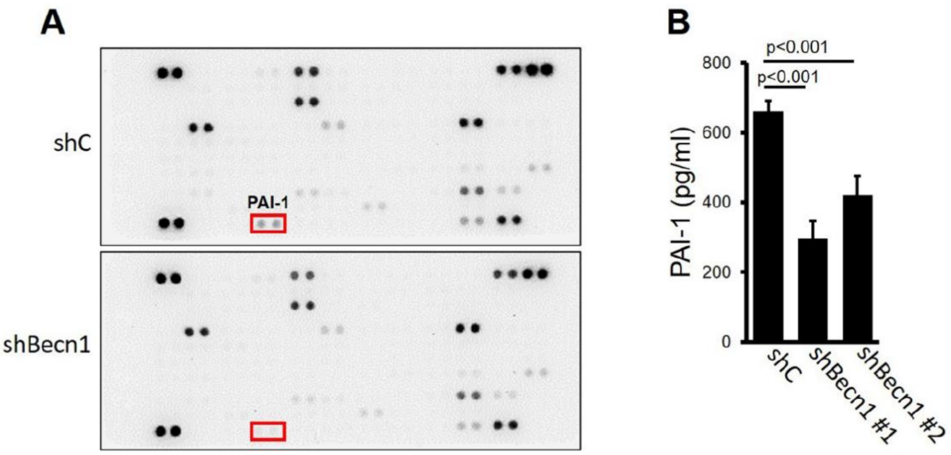

Figure S3. Plasminogen activator inhibitor (PAI)-1 is identified as a cargo candidate for autophagy-mediated secretion. (**A**) Scramble (shC) and shBecn1 B16-F10 cell (shBecn1#1)-derived conditioned media were harvested and screened for soluble factors by an antibody array. (**B**) PAI-1 levels in supernatants from shC and Becn1-knockdown cells (shBecn1#1, and shBecn1#2) were validated by enzyme-linked immunosorbent assay. *p* values were determined by two-tailed Student's *t* test.

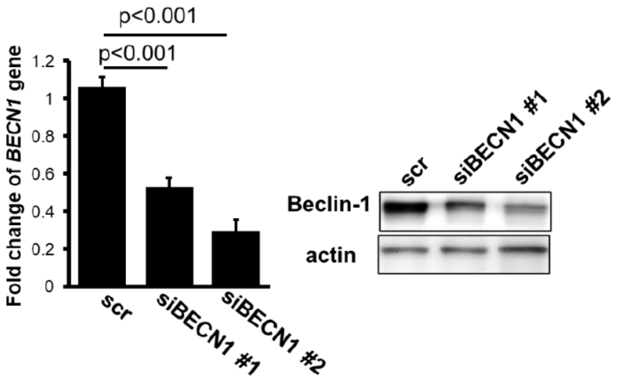

Figure S4. Validation of BECN1 expression in RPMI-7951 cells. RPMI-7951 cells were transfected with RNAi-based oligos targeting scramble (scr) or BECN1 gene (siBECN1#1 and siBECN1#2). Becn1 RNA and protein expression levels were analyzed by quantitative reverse transcription-polymerase chain reaction (left panel) and immunoblotting (right panel), respectively. *p* values were determined by two-tailed Student's *t* test.

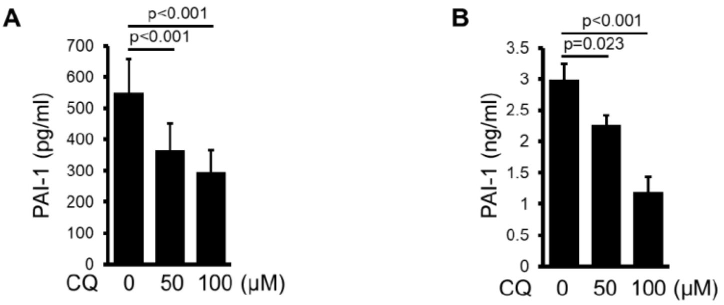

Figure S5. Inhibition of autophagy by chloroquine (CQ) blocks PAI-1 secretion. (**A**) B16-F10 and (**B**) RPMI-7951 cells were treated with CQ at the indicated doses for 16 h. The levels of PAI-1 in supernatants were determined by ELISA assays.

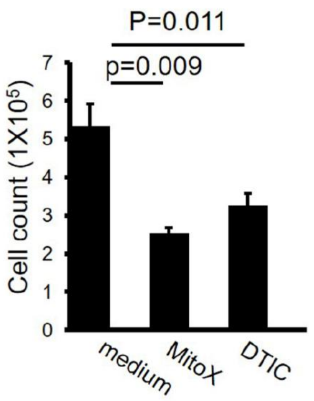

Figure S6. Cell viability in response to chemotherapy. B16-F10 cells were treated with MitoX (2.5 μM) or DTIC (300 μM) for 48 h. Cell number was counted by the Trypan Blue dye exclusion test. *p* values were determined by two-tailed Student's *t* test.

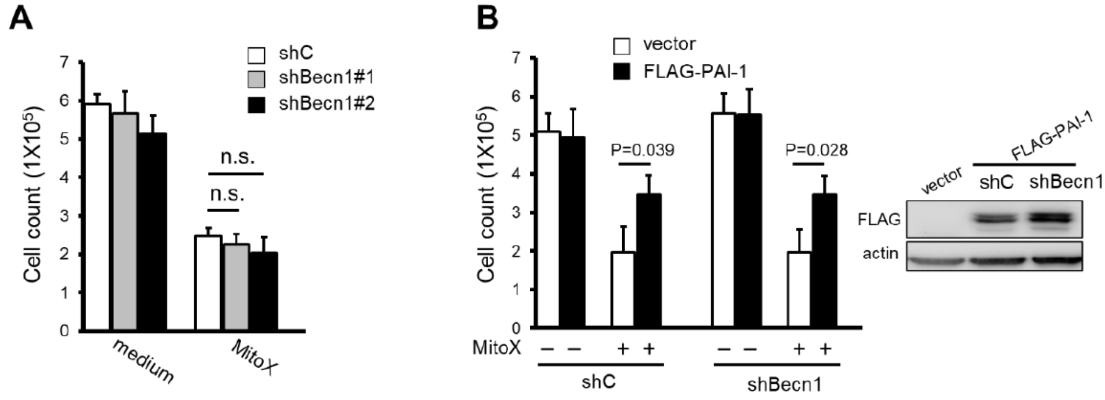

Figure S7. Cytotoxicity of mitoxantrone (MitoX)-treated Beclin1 knockdown and PAI-1 expressing cells. (**A**) scramble-expressed (shC) and Beclin1- silenced (shBecn1#1 and #2) B16-F10 cells were treated with MitoX (2.5 μM) for 48 h. Viable cells were determined by Trypan Blue dye exclusion assay. (**B**) shC and shBecn1#1 with expression of vector or FLAG-PAI-1 constructs were exposed to MitoX (2.5 μM) for 48 h. The cell viability was analyzed by counting the viable cells using Trypan Blue dye exclusion method (left panel). The FLAG-tagged PAI-1 expression was detected by western blotting using anti-FLAG antibody. *p* values were determined by two-tailed Student's *t* test. n.s, not significant.

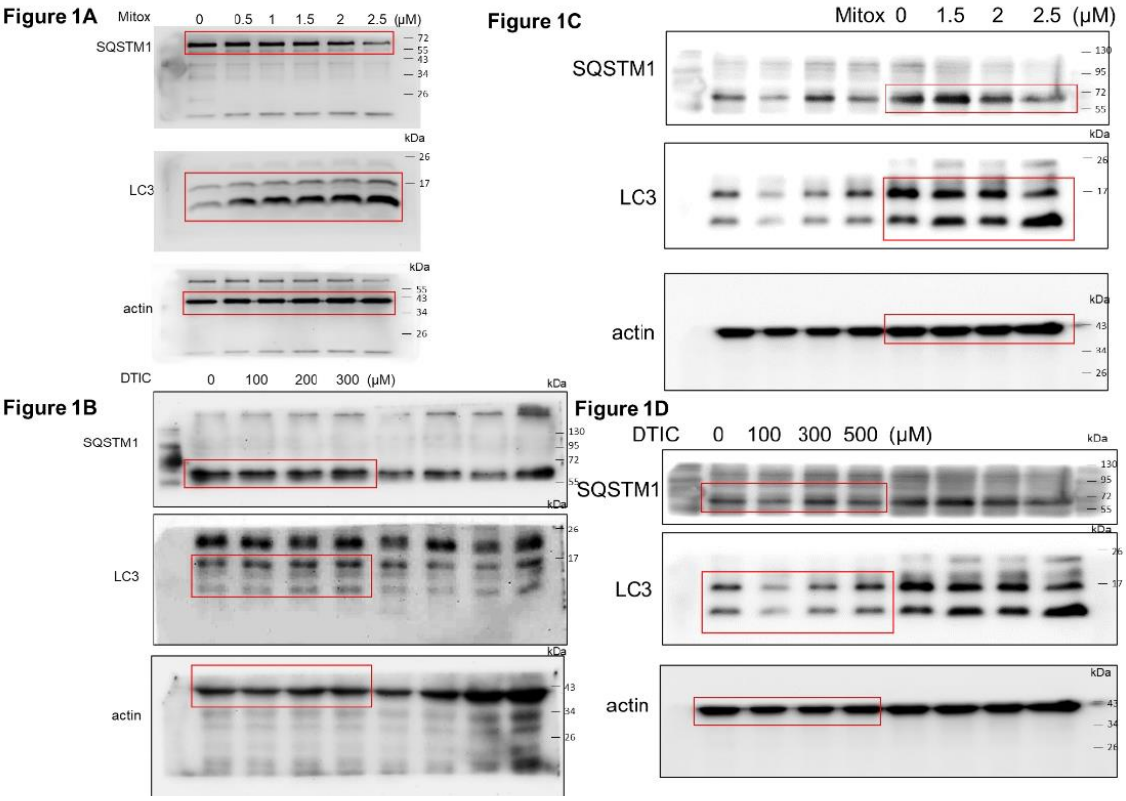

Figure S8. whole western blots of Figure 1.

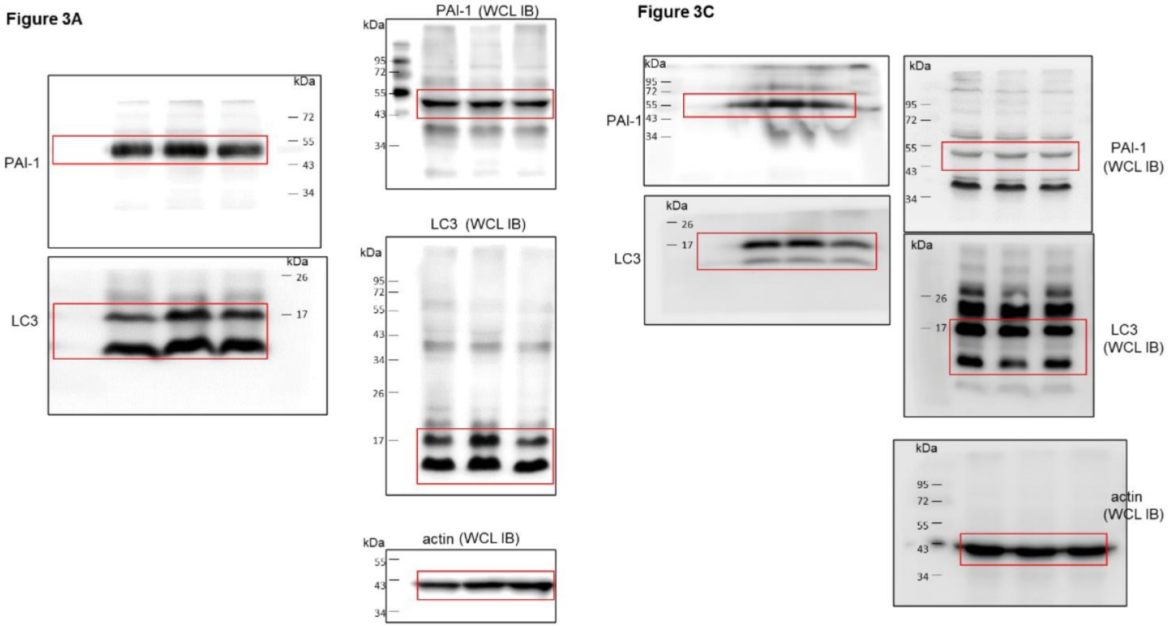

Figure S9. whole western blots of Figure 3.

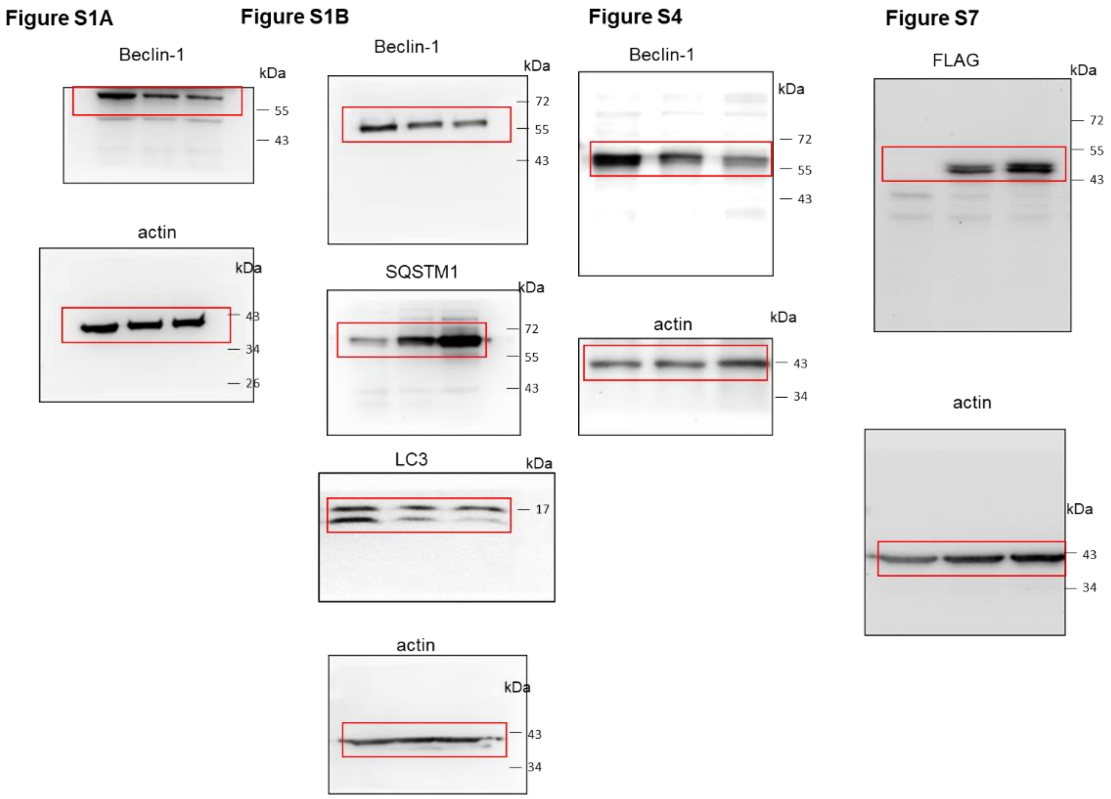

Figure S10. whole western blots of Supplementary Figures.
